# Acetalised Galactarate Polyesters: Interplay between Chemical Structure and Polymerisation Kinetics

**DOI:** 10.3390/polym10030248

**Published:** 2018-02-28

**Authors:** Ionela Gavrila, Patrizio Raffa, Francesco Picchioni

**Affiliations:** Department of Chemical Engineering, ENTEG, University of Groningen, Nijenborgh 4, 9747 AG Groningen, The Netherlands; i.gavrila@rug.nl (I.G.); p.raffa@rug.nl (P.R.)

**Keywords:** sugar-based polyesters, transesterification, galactarate, isopropylidene, methylene, acetal, kinetics, low dielectric constant, stability

## Abstract

In spite of the progress that has made so far in the recent years regarding the synthesis of bio-based polymers and in particular polyesters, only few references address the optimisation of these new reactions with respect to conversion and reaction time. Related to this aspect, we here describe the transesterification reaction of two different acetalised galactarate esters with a model aliphatic diol, 1,6-hexanediol. The kinetics of these two apparently similar reactions is compared, with a focus on the conversion while varying the concentration of a di-butyltin oxide catalyst (DBTO), respectively, the used N_2_ flow-rate. During the first stage of polymerisation, the molecular weight of the end-products is more than doubled when using a 250 mL/min flow as opposed to an almost static N_2_ pressure. Additionally, the resulted pre-polymers are subjected to further polycondensation and the comparison between the obtained polyesters is extended to their thermal, mechanical and dielectrical characterisation. The influence of the acetal groups on the stability of the polyesters in acidic conditions concludes the study.

## 1. Introduction

In a relatively uncertain context regarding the availability of fossil fuels combined with a fast-growing population, and thus also demand, a switch to bio-based materials becomes an attractive solution for the design of sustainable chemical products. Besides the potential of breaking the dependency on finite resources, biomass offers the advantage of being a source of novel building blocks that further on can lead to a broader range and even higher performance polymeric materials.

**Scheme 1:**
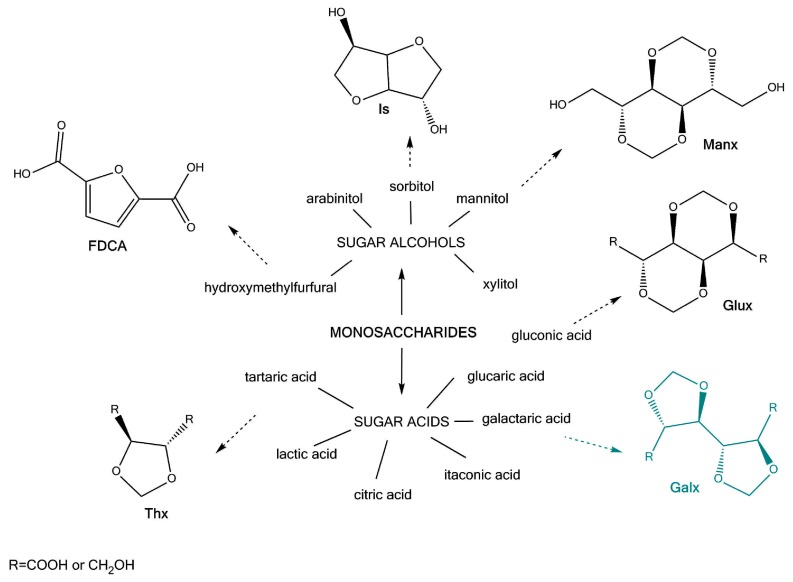
Schematic representation of most commonly used sugar derived cyclic diols and dicarboxylic acids in recent step-growth polymerisation reactions.

What differentiates these building blocks from conventional petroleum-based ones is their readily available functionalisation. Taking carbohydrates as an example, most of the functional groups are hydroxyl and carboxyl, which, besides making the corresponding monomers suitable for step-growth polymerisation, also offers the advantage of further modification of the molecule (by using protecting groups, as shown in Scheme 1). This characteristic comes useful when targeting specific properties in the synthesised polymer. For instance, building blocks derived from glucose, fructose, galactose, mannose, some available at industrial scale, are reported in numerous polymer syntheses suitable for engineering thermoplastics [1,2,3,4,5]. The most well-known and currently commercially available examples are isosorbide and furandicarboxylic acid (FDCA) [6,7,8,9].

Polyesters that are based on FDCA, even though being shown in several studies [9,10,11] to have comparable and even improved properties (such as gas barrier, thermal stability and enzymatic degradation) to the terephthalate homologue, are still in the R&D stage. Similarly, polycarbonates and polyesters based on isosorbide, show remarkable transparency, high *T*_g_ (glass transition temperature) and impact properties [7], and are still under intense research [12]. 

For the here presented acetalised galactarate molecules (the Galx type of structure in Scheme 1) despite their high potential, especially from the availability point of view, few references discuss the characterisation of some derived (co)polyesters [5,13], and little knowledge is built on the optimisation of the polymerisation reaction. 

**Figure 1:**
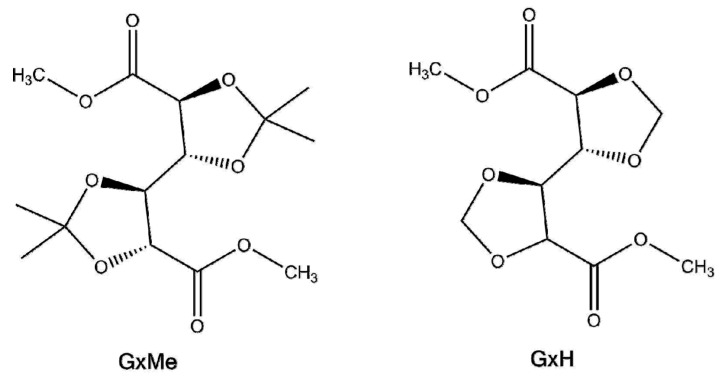
Chemical structure of the two acetalised galactarate esters: dimethyl-2,3:4,5-di-*O*-isopropylidene-galactarate (GxMe) and dimethyl-2,3:4,5-di-methylene-galactarate (GxH).

Overall, looking at the specialty literature, most of the reaction kinetics studies that are available are focused on the synthesis of polyethylene terephthalate (PET). Despite following the same step-growth type of reaction mechanism, it is rather difficult to extrapolate the conditions of the synthesis of PET when using some new (by-cyclic) aliphatic diesters, such as dimethyl-2,3:4,5-di-*O*-isopropylidene-galactarate (GxMe) or dimethyl-2,3:4,5-di-methylene-galactarate (GxH) (Figure 1). Not to be forgotten is also the importance of the structure of the diol co-monomer, which will have an influence on the overall conversion, depending on its reactivity, volatility, thermal stability, etc. For what concerns the catalyst use, for almost any new polyesterification reaction also the metal salt/oxide/alkoxide catalyst will differ depending on its activity for the specific system [14]. Since their incorporation into polyesters is done industrially through bulk polycondensation and most of these molecules are derived from sugars, their thermal sensitivity also needs to be taken into account. 

In this context, prior to an industrial up-scaling of such a polymerisation, it is important to identify the key parameters for the reaction and study their effect on the polyester conversion. This includes the type of catalyst and its optimum concentration, the use of an open/closed system with the aid of inert gas flow for a more effective removal of the formed by-products. 

This study will focus on the above-mentioned effects by looking at the incorporation of a methylene, respectively, an isopropylidene protected dimethyl-galactarate structure into the two-corresponding linear aliphatic polyesters. Since the type of acetal used for making the bicyclic galactarate units varies, the influence of the resulted conformation on the reactivity of the corresponding esters will be discussed as well.

Complementarily, an extensive physico-chemical characterisation of the end products, such as a crystallinity and thermal properties study will be included in order to check for the changes that the acetal structure brings to their morphology and stability.

## 2. Materials and Methods 

### 2.1. Materials

The following chemicals from Sigma-Aldrich were used without further purification: 1,6-hexanediol (>99%), dibutyltin (IV) oxide (DBTO) (98%), citric acid (>99.5%). chloroform, dimethyl sulfoxide, methanol and diethyl ether were HPLC or technical grade. 

The dimethyl-2,3:4,5-di-*O*-isopropylidene-galactarate (GxMe) (>99%) and the dimethyl-2,3:4,5-di-methylene-galactarate (GxH) (>99%) were received from Royal Cosun and used without further purification.

### 2.2. Synthesis Procedure

An equimolar mixture of GxMe or GxH di-ester, respectively, and 1,6-hexanediol, together with an amount corresponding to 0.3–4 mol % (to the ester) DBTO, were brought into a 3-neck cylindrical flask equipped with a magnetic coupling stirrer, a nitrogen gas inlet, and a temperature-controlled distillation setup. Prior to the reaction, nitrogen is flushed through the reaction. During the reaction a controlled N_2_ flow was used, set at either 30 mL/min or 250 mL/min.

The temperature of the reaction was set and maintained at 142 ± 2 °C (temperature of the oil bath) and a constant nitrogen flow was set during the first stage of the reaction (for 3.5 h). Subsequently, the distillation setup was replaced with a vacuum adapter and high vacuum was applied (0.01 mbar). The progress of the reaction was followed by monitoring the torque values while keeping the temperature constant. 

The resulted product is dissolved in chloroform and after precipitated and was thoroughly rinsed with diethyl ether. The polymer powder is recovered by vacuum filtration and dried of solvent until there constant weight in the vacuum oven.

### 2.3. Equipment and Technique

To confirm the structure of the obtained polyesters, NMR (Nuclear Magnetic Resonance) and FTIR (Fourier Transform Infrared Spectroscopy) analysis methods were used.

The NMR spectra were recorded using a Varian Mercury Plus equipment operating at 400 MHz. Approximately 10 respectively 50 mg of sample were dissolved in 1 mL of deuterated chloroform for 1H NMR and 13C NMR. A total of 64 scans were acquired for the 1H and 2000 for the 13C spectra, with a relaxation delay of 1 s.

For the kinetic study, samples of the reaction mixture were taken at different intervals of time. For this, a Teflon tube connected to a syringe was hermetically attached to the polymerisation setup (so that no air can protrude). At regular time periods, a polymer sample is drawn into the tube, weighed, and subsequently dissolved in chloroform. The prepared solutions are further used for determining the molecular weight. The conversion is calculated by using Carothers equation for linear polymers for equimolar used monomers:

(1) $$p=1-\frac{1}{X_{n}};$$

where *Xn* is the degree of polymerisation.

The number (*M*_n_) and weight (*M*_w_) average molecular weight and dispersity of the polymer samples were determined at 35 °C using a Viscotek Size Exclusion Chromatography system. Triple detection was used (light scattering, viscometer, refractive index). The attached columns were two Agilent PLgel 5 µm MIXED-C, 300 mm, and a guard column Agilent PLgel 5 µm Guard, 50 mm. Chloroform was used as mobile phase set at a flow rate of 1 mL/min and the molecular weight values of the samples were determined based on a universal calibration method. The calibration was made on narrow dispersity polystyrene standards (Agilent Technologies Netherlands B.V.) that were in the range of 645–3,001,000 g/mol. The integration of the peaks was done at a fixed dn/dc value. 

Thermo-gravimetrical analysis was carried out in a Mettler-Toledo analyser (TGA/SDTA851e). Samples of approximatively 5 mg were pre-weighed in alumina crucibles. The weight loss of the polymers was monitored, while the temperature was increased from 30 to 500 °C at a heating rate of 10 °C/min under 80 mL/min. flow of Ar.

For the thermal characterisation of the polymer samples, a Perkin–Elmer Differential Scanning Calorimeter Pyris 1 was used under a N_2_ atmosphere. The heating rate was set to 10 °C/min, and an amount of approximately 5 mg of sample was heated from −20 °C to 200 °C. The glass transition temperature (*T*_g_) values were taken as midpoint for the inflection of the second heating cycle. 

Wide-Angle X-ray Diffraction (WAXD) spectra were measured in the 5–60° range on both powder and polymer films by using a Bruker D8 Advance Diffractometer (Cu radiation, λ = 1.5418 Å).

The contact angle measurements were done using a DataPhysics OCA—Series equipment. The value of the angle of the drop of demineralised water onto the polymer films was calculated as average of seven drops of approximately 10 μL volume on different spots on the film surface. 

For the tensile test, thin polymer films were prepared by casting. Approximately 200 mg of polymer sample were dissolved in 1 mL of CHCl_3_ and the solution was casted on a Teflon plate. The vacuum dried resulted films were cut into rectangular strips 5 mm wide. Prior to the test, the thickness of the films was measured with a micrometer screw gauge. The measurement was performed at room temperature on a Instron 5565 device, Series IX. The crosshead speed was set at 26 mm/min.

For determining the dielectric constant values, rectangular plates of 56 × 64 × 0.5 mm size were compressed moulded at a temperature of 140 °C for 30 min and subsequently quenched. The dielectric characterisation was done externally at ASTRON, Netherlands Institute for Radio Astronomy, by using a Substrate Measurement System of AC Microwave. The exposure to the electromagnetic field consists in using a ring resonator with the frequency range set between 1 and 10 GHz.

For the hydrolytic stability test, thin polymeric films were prepared similarly to the ones used for the tensile test. Pre-dried polymer films were immersed and left in a 5% citric acid water solution at different temperatures: 25, 50, and 75 °C. Samples were taken after several days of treatment, rinsed several times with demi-water, dried overnight in the vacuum oven, dissolved in CHCl_3_-d, and then analysed with 1H NMR. 

## 3. Results and Discussion

### 3.1. Mechanism of the DBTO Catalysed Transesterification

For transesterification, similarly to direct polyesterification, the use of acids catalysts is needed for obtaining high molecular weight polymers. However, for the former due to a slower rate of the hydroxy-ester interchange, the use of a catalyst is imperative. Even if data about the activity of different types of used organometallic derivatives (mostly Lewis acids) is available, it is easily noticed that the catalysts efficiency for similar reactions differs substantially. For instance, the use of Ti(OBu)_4_ even though it is favourable for synthesizing PBT, induces discolouration in PET through various side reactions, such as the ones that generate diethylene glycol [15]. Furthermore the efficiency of the same catalyst for the reaction of GxH and C4–C12 linear aliphatic diols, was reported to be significantly lower than of DBTO [15,16]. 

In this context, it is understandable that the mechanism pathways for the transesterification reaction can change depending on the used catalyst-substrate combination. Nevertheless, for the reactions that are explored in the current study the focus is on the mechanism that involves DBTO. 

**Scheme 2:**
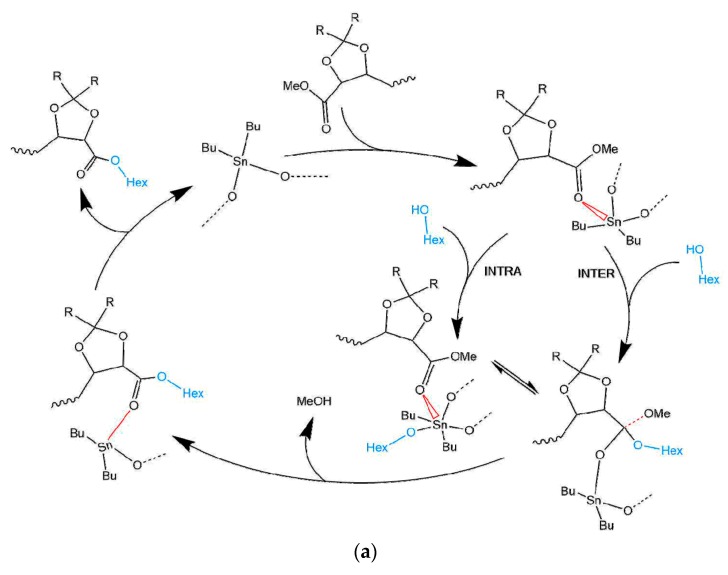

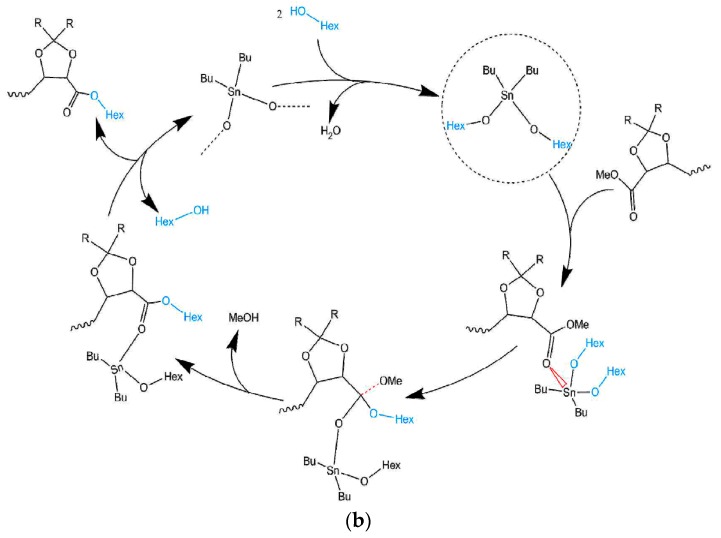
Proposed mechanism pathways for the dibutyltin (IV) oxide (DBTO) catalysed transesterification: Lewis acid inter-and intra-molecular nucleophilic attack (**a**) and exchange/insertion mechanism (**b**). Adapted from ref. [17,18].

Recently, two possible mechanisms were postulated for transesterification reactions catalysed with organo-tin complexes [17]. They both are based on the distinguishable characteristic of the Sn atom of being able to extend its coordination number, which allows for the coordination of extra ligands and/or associative ligand exchange. Based on this, there is some agreement that depending on the structure of the alcohol, the mechanism of the transesterification will follow either a classical Lewis acid path where Sn coordinates to the ester group activating the carbonyl carbon and thus favouring a nucleophilic attack of the alcohol, or it will follow an exchange/insertion pathway, as depicted in Scheme 2. The latter is in particular the generally accepted route for Sn catalysed transesterifications where a ligand exchange with the diol is unlikely to happen. As shown in Scheme 2b, it implies first the formation of a tin alkoxide, which will coordinate to the ester, insert the carboxyl group, and then exchange again to reform the intermediate catalytic species. 

As supporting data for the exchange/insertion mechanism Hu et al. evaluated several reactions of tin based catalysts with an aliphatic alcohol [15]. Their findings indirectly indicated that the tin catalysts with the highest activity for their transesterification reactions were the ones that can also form a dialkoxide with the diol used in the ester-hydroxy interchange. That implicitly leads to the fact that the overall rate will be dependent on the formation of the intermediate product. Thus DBTO in the case of transesterification of polyethylene-*co*-(vinyl acetate) with 1-octanol was found to accelerate the reaction as when compared with Zn acetate or dibutyltin sulfide [15]. Similar results were also reported for an R_2_Sn oxide (including DBTO) catalysed reaction of propylacetate with methanol [19].

#### 3.1.1. Effect of the N_2_ Flow Rate

**Scheme 3:**
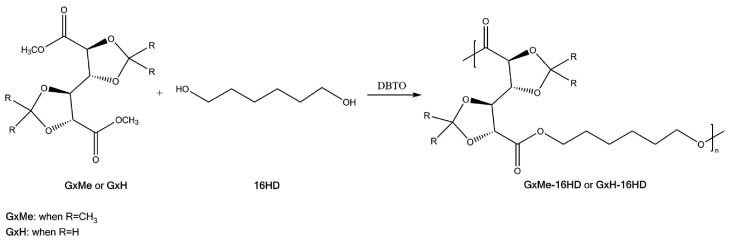
General synthesis reaction of the GxMe-16HD, respectively, GxH-16HD homopolyester.

A series of GxMe and GxH based homopolyesters were synthesized through polymerisation in melt, as depicted in Scheme 3. The chosen procedure was based on a similar one reported by Lavilla et al. [16].

The interest was to investigate whether the reaction time can be optimized. Two parameters were considered varying in order to study their effect on the transesterification reaction: the flow-rate of nitrogen gas and the catalyst concentration. The two differently acetalised galactarate esters were compared for their (possible) effects on the transesterification conversion. Sampling from the reaction was started only after 30 min when the monomers were already converted into (short) oligomers.

**Figure 2:**
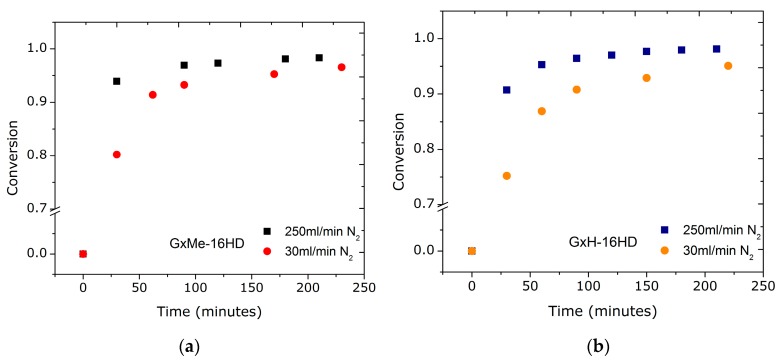
Conversion plots for the transesterification reactions of GxMe and GxH with 1,6-hexanediol when using a 30 mL/min (**a**), respectively, a 250 mL/min N2 flow-rate (**b**).

Since using a gas flow through the polymerisation system can induce a faster mass transport not only of the formed methanol but also of the unreacted monomer (in the beginning of the reaction) there were concerns on the efficiency of having a higher N_2_ flow, and thus on the perturbation of the equimolar ratio. Nonetheless, as shown in Figure 2, the trend proved to be similar for both transesterification reactions, a conversion higher than 85% being achieved in the first 30 min for the reaction when using the highest flow of 250 mL/min. Accordingly, for the reduced flow of 30 mL/min, 75–80% conversion was obtained. This further indicates that the effect relies on the mass transfer of solely the by-product and that there is a relation between the flow-rate and conversion independent of the chemical structure of the monomers. For similar transesterification reactions, mostly the use of a N_2_ environment at atmospheric pressure is reported, however, for reactions where the melt viscosity is expected to be high, a N_2_ bleed through the reactor is used (without any specification on the exact flow rate) [20].

These findings for the reduced N_2_ flow, according to which both reactions of GxMe and GxH with 1,6-hexanediol are characterised by slower kinetics, clearly depict the negative effect of an inefficient removal of the by-product on the overall rate of the reaction. This was also visually observed as a delayed formation and condensation of methanol through the distillation column connected to the reactor.

In few other studies where the kinetics of interchange reactions is discussed, the influence of the diffusion of the condensation by-product is considered. Davis and Pell [21] looked at the effect on the conversion of PET when using different thicknesses of the melt and conclude that in melts that are thicker than 0.03 cm, the polymerisation becomes diffusion dependent. As also shown here, the role of purging a stream of dry inert gas is mentioned often with a positive effect on the removal of the condensate, however without any quantification [22,23,24,25]. 

**Figure 3:**
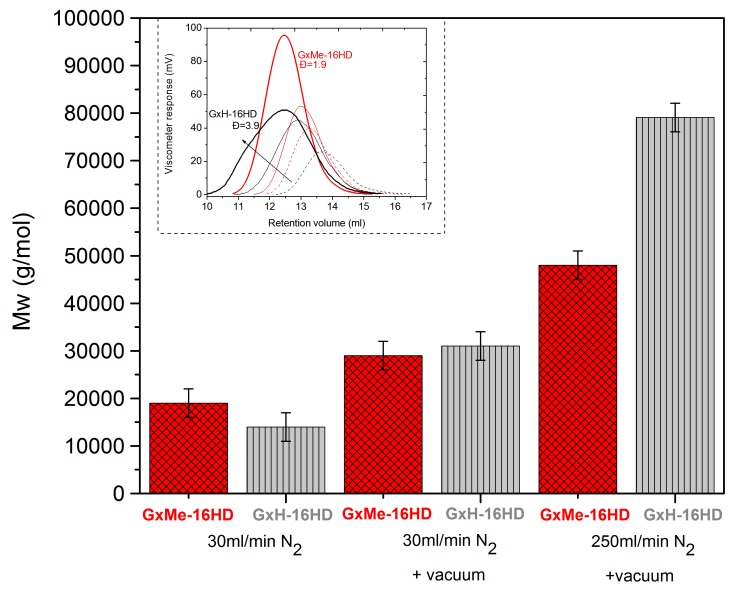
*M*_w_ values comparison and GPC distribution curves (in the insert) of GxMe-16HD and GxH-16HD after 3.5 h under 30 mL/min N_2_ (dot line); after 3.5 h under 30 mL/min N_2_ + 0.5 h at 10^−2^ mbar (solid line); after 3.5 h under 250 mL/min N_2_+ 0.5 h at 10^−2^ mbar (bold solid line). Error bars are taken here as minimum and maximum value (samples were made in duplicates).

It could be argued that even if delayed in the early stages of the polymerisation, the shorter formed oligomers can still be further condensed under high vacuum conditions, and thus the molecular weight will still be significantly increased. This aspect was also checked and it was noticed that despite a doubling in the molecular weight to *M*_w_ = 30,000 g/mol after 30 min at 0.01 mbar vacuum, when compared to the conversion of the samples that were obtained during the 250 mL/min N_2_ flow stage, this was still significantly lower (Figure 3).

During transesterification, no major difference was found in conversion when comparing the isopropylidene acetalised sample with the methylene acetalised one. The molecular weight of the samples at the end of transesterification is comparable. However, after switching to vacuum, in the case of GxH-16HD a stronger increase in viscosity of the melt is observed making impossible to continue stirring the reaction for more than 30 min without increasing the temperature. This observation was in line with the sudden broadening of the molecular weight distribution curve (Figure 3). The dispersity for this sample was determined to be 3.9. The same tendency in a broader dispersity was noticed for all GxH-16HD reactions after applying vacuum.

#### 3.1.2. Variation of the Catalyst Concentration

Besides the effect of a flow of N_2_ through the reaction setup, the role of the catalyst concentration was also investigated. The used concentration of DBTO for both types of reactions was varied between 0.3 and 4 mol % with respect to the GxMe, respectively, GxH ester. This concentration range was chosen in order to determine the optimum catalyst load based on the hypothesis that an increase in catalytic active sites will shorten the reaction time [25,26]. 

Furthermore, based on the results that were obtained when varying the gas flow-rate, the transesterification reactions were performed under a flow of 250 mL/min N_2._

**Figure 4:**
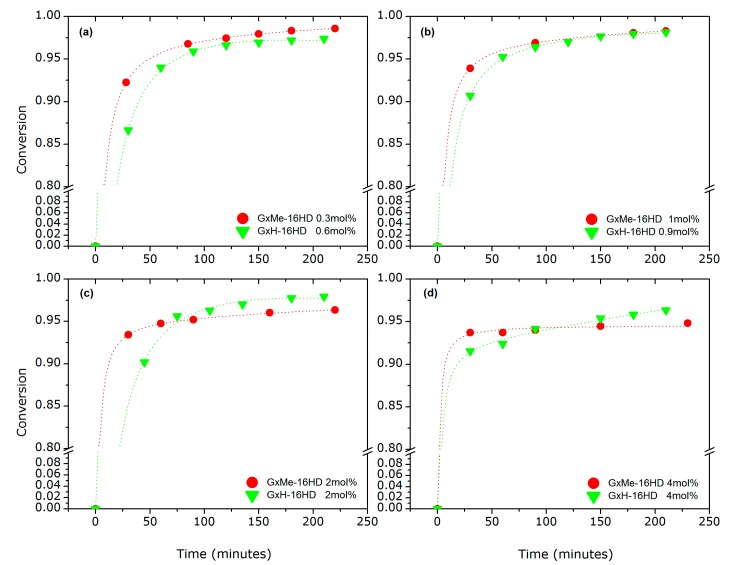
Conversion plots for the transesterification reactions of GxMe respectively GxH with 1,6-hexanediol when varying the DBTO concentration: 0.3–0.6 mol % (**a**); 0.9–1 mol % (**b**); 2 mol % (**c**); 4 mol % (**d**).

The determined conversions for these samples that were taken at different time intervals are depicted in the graphs in Figure 4. It was noticed that the reactions, independent of the catalyst concentration, proceed relatively fast, reaching a conversion higher than 85% in the first 30 min. In the first 5 min of the reaction at 140 °C vapour condensation on the distillation side arm of the reactor was noticed. Based on the proposed mechanism (Scheme 2), the condensate is assumed to be methanol. Even though the trend is characteristic for step-growth polymerisation, this indication of fast kinetics was unexpected when compared with conversion plots for the same catalyst used in other similar reactions [15,17] or even for other catalysts used in the synthesis of PET and PEN [27]. 

Additionally, the conversion trends for different concentrations of DBTO showed to be distinct for the two types of acetalised polyester. At all of the tested catalyst concentrations, the initial kinetic rate is higher for the reaction of the isopropylidene acetalised galactarate ester. Thus, the expectation that the activation of the carbonyl group would be faster for the GxH esters due more spacing around the carbonyl compared to GxMe seems not to be supported by these results. 

At longer reaction times differences in the conversion profile are observed, dependent on the catalyst concentration. For the low DBTO concentrations (0.3–1 mol %) after approximately one hour, the conversion of both tends to plateau around 97–98% (Figure 4a,b). 

For the higher concentrations of catalyst, an overall negative effect on the conversion with a higher profile for the GxH-16HD samples was observed. 

Moreover, a threshold in the concentration of catalyst also seems to play a role. When comparing the conversion as function of the catalyst concentration for the two acetalised galactarate polyesters, the lower conversion profile when increasing the load of DBTO is more visible for the GxMe-16HD samples (Figure 4c,d). Returning to the proposed reaction mechanism, two centres are possible for the Sn catalyst coordination: the favoured one, the oxygen from the ester carbonyl, but also the one from the acetal groups. At low concentrations of DBTO, the transesterification reaction is favoured, however, when increasing the amount of catalytic species, depending on the type of acetal group, catalyst can be coordinated there as well. The effect is more likely to happen for the GxH type of protection group, due to no hindrance as opposed to the GxMe acetal that has two methyl pending groups. This hypothesis would explain also the broadening of the dispersity for the GxH polyester samples after longer reaction times.

#### 3.1.3. Analysis of the Kinetics of the Transesterification Reactions

Based on the findings discussed above, especially on the significant effect of N_2_ flow-rate on the conversion, the data included in the calculations for the overall kinetics of the transesterification correspond to the reactions that were performed under a 250 mL/min N_2_. This also supports one of the conditions that are established beforehand for simplification purposes, that the reaction of the galactarate ester unit and 1,6-hexanediol is to be considered irreversible.

Based on the premise that the reaction is second order [14,28] and by including the change in catalyst concentration in the rate constant, *k_a_′*, the chemical rate equation can be defined as:

(2) $$-\frac{d\left[Gx\;\right]}{dt}=k_{a}\prime \;\left[Gx\right]\left[16HD\right]$$

Furthermore, due to the change in volume with conversion (with the removal of the formed methanol), a correction factor for the monomer and catalyst concentration was considered. 

The resulted monomer concentration will be expressed as:

(3) $$\left[Gx\right]=\frac{{\left[Gx\right]}_{0}\left(1-p\right)}{V_{0}-\left(V_{0}-V_{f}\right)\;p}$$

and the catalyst concentration as:

(4) $$\left[cat\right]=\frac{V_{0}}{V_{0}-\left(V_{0}-V_{f}\right)\;p}$$

where: [*Gx*]_0_ = the initial concentration of monomer; *V*_0_ and *V_f_* stand for the initial respectively final volume of the reaction product.

Conversion, *p*, is calculated using Carothers equation:

(5) $$p=\frac{{\left[Gx\right]}_{0}}{\left[Gx\right]}=\frac{1}{1-X_{n}}$$

where: *Xn* = the degree of polymerisation.

Since the stoichiometric ratio, *r* = 1 and $r=\frac{\left[Gx\right]}{\left[16HD\right]}$, the rate equation can be rewritten as:

(6) $$-\frac{d\left[Gx\right]}{dt}=k_{a}\prime {\left[Gx\right]}^{2}$$

where *k_a_*′ is the apparent kinetic rate.

The concentration values of the galactarate esters, [*Gx*], include the correction factor for the change in volume of the reaction mixture due to sampling and conversion:

(7) $$\left[Gx\right]=\frac{\left(1-p\right){\left[Gx\right]}_{0\;}}{V_{0}-\left(V_{0}-V_{f}\right)p}$$

By integrating relation (6) =>

(8) $$\frac{1}{\left[Gx\right]\;}=\frac{1}{{\left[Gx\right]}_{0}}+k_{a}\prime \;t$$

By plotting the values as a function of the transesterification time a linear trend of the 1/[*Gx*] is expected which would indicate the second order of the overall reaction. 

Most of the authors agree on a second order reaction in the case of catalysed transesterification [14,28]. Even so, the fitting of the data is not always accurate due to parameters which are not included the kinetic study: such as the use of a closed system (leading to incomplete removal of the formed by-product) ignoring the change in volume with conversion or sampling or even not considering the effect of catalyst concentration [29].

**Figure 5:**
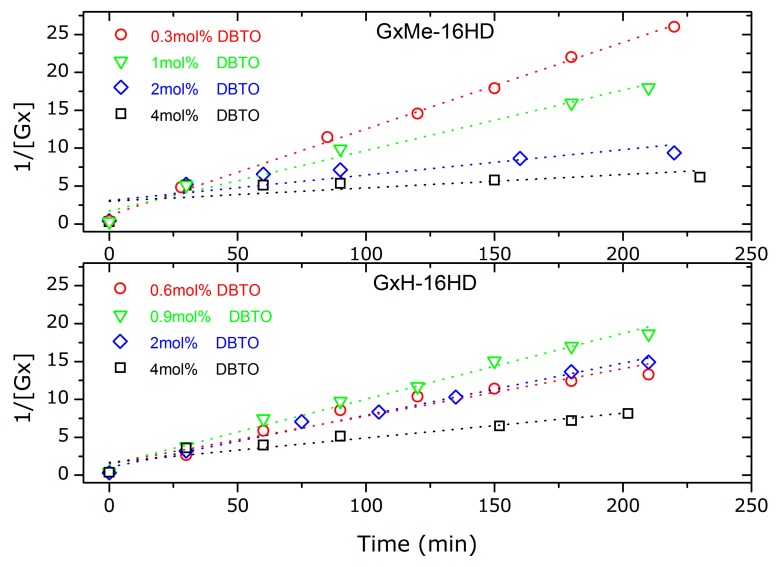
Kinetic plots for second order fitting of the GxMe-16HD, respectively, GxH-16HD transesterification reactions at different DBTO concentration: 0.3, respectively, 0.6 mol %; 0.9 respectively 1 mol %; 2 mol %; 4 mol %.

The catalyst load seems to determine the transesterification kinetic rate and thus linearity of the data appears to be sensitive to the amount of DBTO, as shown in the kinetic plots in Figure 5. In particular, the fit is found to be linear for the samples that also showed the highest conversion: for GxMe-16HD catalysed with 0.3 mol % DBTO and GxH-16HD with 0.9 mol % DBTO. For the reactions catalysed with higher concentration of DBTO samples, the kinetic rate slope shows a deviation from linearity after the first 30 min of the reaction (where the conversion is already higher than 85%). This effect was observed before i.e., in the case of the acid catalysed reaction of adipic acid with diethylene glycol. Nevertheless, a possible explanation in that case was connected to the change in polarity with conversion, from the acid to the ester groups [30]. 

In this case, the nonlinearity for the reactions with higher DBTO concentration can also be attributed to the presence of secondary reactions, as mentioned before [29]. No similar response was found reported in literature when using DBTO or other Lewis acids for catalysing similar reactions However, since the lowest used DBTO concentration already determines conversion higher than 85% after 30 min, it could be argued that the excess of catalyst in the presence of even small traces of methanol will favour the depolymerisation reaction, and thus the equilibrium back towards the monomers. Similarly, the sensitivity of some catalysts to traces of water has been reported before, i.e., Ti (IV) butoxide showed improved activity when it was added at later stage in the reaction and not during the direct esterification (which involves water formation as the by-product) [25].

The deviation from a linear fitting for a second order reaction is reflected in the R squared values depicted in Table 1. The best fitting (R^2^ = 0.996) was found at the lowest catalyst concentration, of 0.3 mol % for GxMe-16HD. Contrary, for the methylene protected polyester, the best linear fitting, R^2^ = 0.988 and R^2^ = 0.986 was for higher catalyst concentration of 0.9 mol % and 2 mol %.

### 3.2. Thermal and Mechanical Characterisation

As also reported previously by Lavilla et al., the GxH-16HD homopolyester shows superior thermal stability and tensile strength when compared to homologous linear aliphatic polyesters [31].

**Figure 6:**
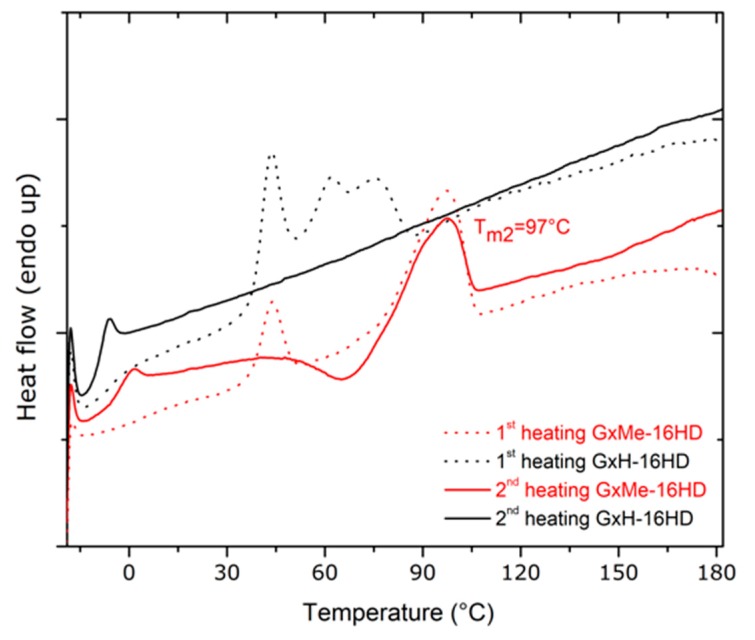
DSC curves of GxMe-16HD and GxH-16HD displaying the melting behaviour during the first and second heating cycle when heating with 10 °C/min.

The GxMe ester unit however besides the same semi-rigid bicyclic structure brings some more free volume due to the pending methyl groups of the isopropylidene acetal protection. A higher *T*_g_ is determined by intermolecular interactions between the polymeric chains that have a low internal mobility. Thus, a decreased free volume with the presence of stiff monomeric units as in the case of the GxMe-16HD homopolymer was expected to have the highest *T*_g_ between the two. The DSC analysis samples comes in good agreement showing a 5 °C higher *T*_g_ for the latter. Connected to the difference in *T*_g_, a melting region was identified for both of the samples in the first heating run, but only for the GxMe-16HD during the second heating cycle (Figure 6). 

In order to confirm whether this faster crystallisation is independent of the thermal history, samples of the GxMe-16HD and GxH-16HD polyesters were kept at 75 °C for 24 h under N_2_ and the DSC analysis was repeated. A melting region was again observed for the GxMe-16HD during both scans, but still not for the GxH-16HD sample (supporting information Figure S2). Similarly, when thin films were prepared by solvent casting, it was noticed that the GxMe-16HD samples became opaque immediately after the solvent evaporation as for the GxH-16HD, the films remained almost fully transparent.

**Figure 7:**
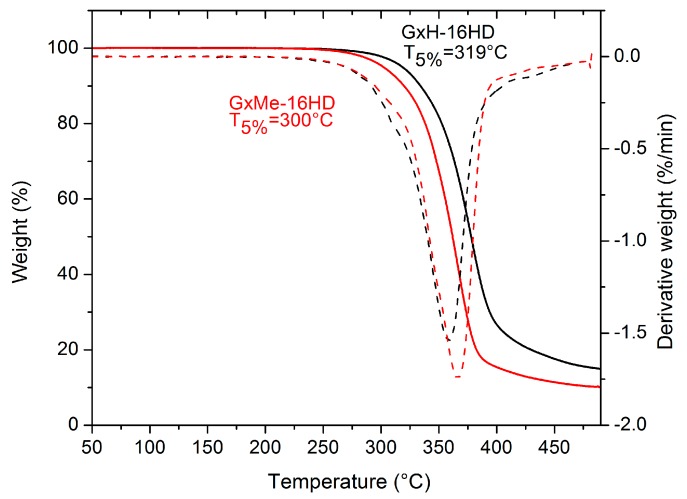
Weight loss of GxMe-16HD and GxH-16HD determined by thermo-gravimetrical analysis (TGA).

The type of structure of the acetal protection was shown also to have a different role in the thermal stability of the corresponding polyesters as represented in Figure 7. Both homopolyesters are stable up to more than 250 °C. Also, from the 5% weight loss temperature, a shift of 15–20 °C can be observed between the two, with a lower decomposition rate being linked to the higher thermal stability of the GxH based polyester.

Complementary to the DSC analysis, the crystallisation patterns of the homo-polyesters were identified through X-ray diffraction. As known from literature for the GxH aliphatic polyesters, the crystallisation tendency increases with the length of the diol, being the lowest when comparing a 6C with a 12C linear aliphatic diol. Furthermore, it was noted that the GxH-16HD polyester is less crystalline and even showing a negative effect on crystallinity when compared with homologous non-cyclic polyesters [16]. This can be related to the fact that despite the rotation freedom from the bond between the two acetal units, the acetal structure itself due to its bulkier nature induces a broader distribution of spherulites [32].

Based on these observations, it could be expected that the pending methyl groups from the GxMe acetal groups will show an even more pronounced hindrance effect with respect to the chain packing density. However, since these side groups are short, they favour some stereoregularity of the chain, and thus, the formation of better defined crystallite regions (as also revealed by the WAXD analysis). The two polyesters display completely different diffraction patterns. GxMe-16HD is characterised by one major diffraction peaks at 19° with a smaller one at a 10° theta angle while GxH-16HD displays several smaller peaks and shoulders, the most intense ones in the 21–23° region. That is in good agreement with the observed transparency/opacity.

**Figure 8:**
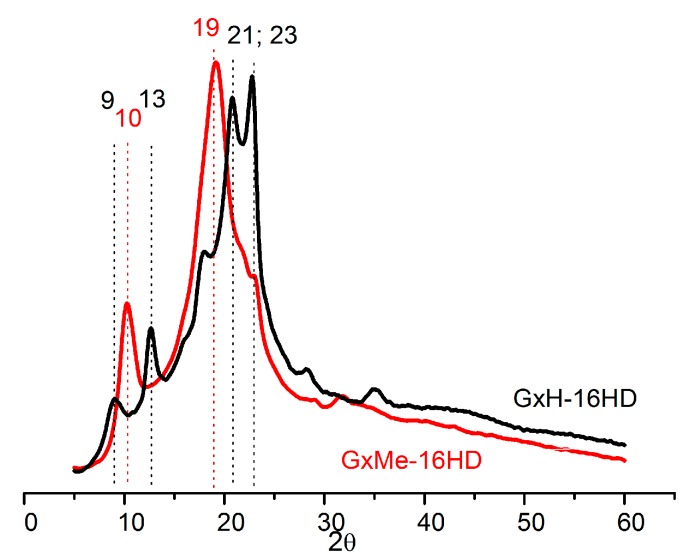
Wide-Angle X-ray Diffraction (WAXD) spectra of GxMe-16HD and GxH-16HD after pre-annealing at 75 °C.

Nevertheless, as shown in Figure 8, both types of sample are characterised by relatively broad peaks, overall indicating a more prominent amorphous behaviour combined with the presence of small crystallites. 

The relative crystallinity was calculated based on the diffracted intensity of the amorphous, respectively, crystalline phases determined from the WAXD analysis. 44% relative crystallinity was determined in the case of GxMe-16HD and 35% for the GxH-16HD when the analysed samples were kept in advance at 75 °C for 24 h. 

Resistance to deformation under stress characterised by the Young’s modulus was also found to be slightly higher for GxMe-16HD (Table 2). The more ordered and packed arrangement of the chains hinder the sliding of the chains, and therefore determine a stiffer response than of the less crystalline GxH-16HD samples.

### 3.3. Dielectric Constant Characterisation

Besides the thermal and mechanical properties, one of the aims of the study was also checking the influence of the type of acetal structure on the dielectric response of the corresponding polyesters. What determines a low dielectric constant of a material is a small polarisation response when an electromagnetic field is applied to it. Since the dielectric constant and the loss dispersion is dependent on the frequency of the applied field it is worthwhile mentioning the fact that the response of the studied polyesters was analysed at a varying frequency between 1 and 10 GHz. 

Due to its additivity property, it was possible to calculate the dielectric constant values by means of the molar polarization group contribution [34]. The latter is defined for isotropic polymers as:

(9) $$P_{LL}\;\frac{\varepsilon -1}{\varepsilon +2}V\;{\mathrm{cm}}^{3}/\mathrm{mol};$$

where *V* = molar volume and ε = dielectric constant (or relative permittivity).

Due to the dependence on the molar volume, the group contribution calculation method that was used in this case, as shown in Table 3 allows for the differentiation between amorphous and semicrystalline materials. Nonetheless, the determined permittivity values do not show a significant variation. When comparing the GxMe-16HD and GxH-16HD, a significantly lower permittivity resulted for the former. 

**Figure 9:**
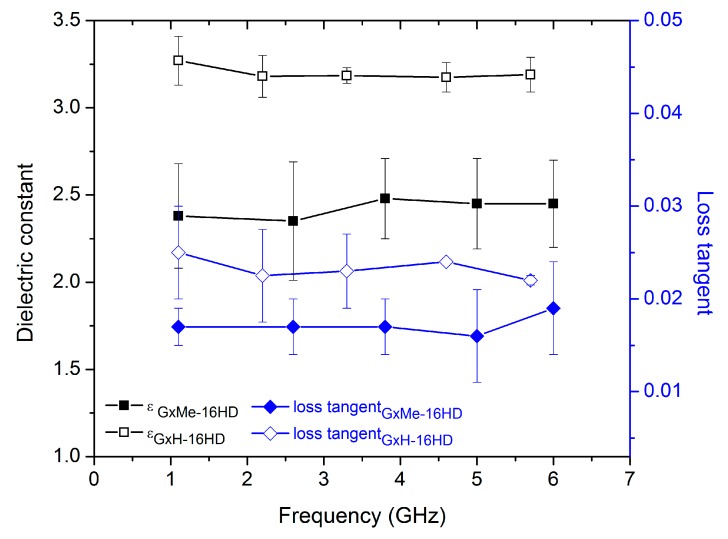
Relative dielectric constant and loss tangent of GxMe-16HD and GxH-16HD samples determined as function of a frequency sweep between 1 and 7 GHz.

For the experimental determination, the prepared polyester plates with predefined geometry were subjected to an electrical field of variable frequency. The obtained average values (Figure 9) proved to be in good agreement with the ones that were estimated with the group contribution method, emphasizing the positive effect of the GxMe isopropylidene acetal group on decreasing the overall relative permittivity. As it is already known, the presence of pending groups, in this case the methyl groups of GxMe-16HD determine a higher molar volume and thus more spacing at the structure level, which further on translates into a lower polarization response of the material [35,36].

Despite the deviation in the determined dielectric constant values, assigned to the variation in the thickness of the polyester plate samples, no significant influence from the crystallinity density was found (as also shown from the theoretical group contribution calculations). A similar finding was reported also for PEEK amorphous and up to 40% semicrystalline [37].

### 3.4. Hydrolytic Stability

Resistance to acidic conditions of the synthesised polyesters was tested by immersing thin polymer films in a pH = 2 citric acid solution. Samples were tested at room temperature, but also at 50 and 75 °C. 

The samples that were kept at 25 °C showed no visible sign of degradation, however when increasing the temperature, the polymer films became more brittle. The GxH-16HD samples that were treated at 75 °C could not be recovered from the acidic solution. It was relevant to investigate whether the physical degradation of the samples was caused by a deacetalisation and/or ester hydrolysis. A comparison of the 1H NMR spectra of the polyesters before and after the citric acid treatment shows the stability of both types of acetal protection (peaks in the 1.3–1.5 ppm region for GxMe acetal and, respectively, in 5.1–5.3 ppm region for the GxH acetal) throughout the study. Similarly, it was reported before that the methylene protection is more stable in highly acidic environment than the isopropylidene [38]. Even though unrelated, it is remarkable from an application point of view that the thermal stability of the two acetalised galactarate esters and implicitly of the derived polyesters, is reverse to the hydrolytic stability. 

**Figure 10:**
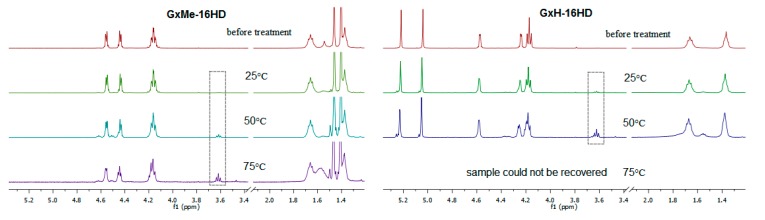
1H NMR overlay spectra of GxMe-16HD and GxH-16HD samples before and after hydrolytic treatment in citric acid 5% solution for 30 days at 25, 50, and 75 °C.

Furthermore, as depicted in Figure 10, in the 1H NMR spectra, the apparition and evolution of the peak at 3.7 ppm was observed and was assigned to correspond to the newly formed CH_2_ group that was connected to a hydroxyl end-group as consequence of the ester cleavage. Based on its quantification, the kinetics of the cleavage of the ester bonds were followed as function of temperature.

**Figure 11:**
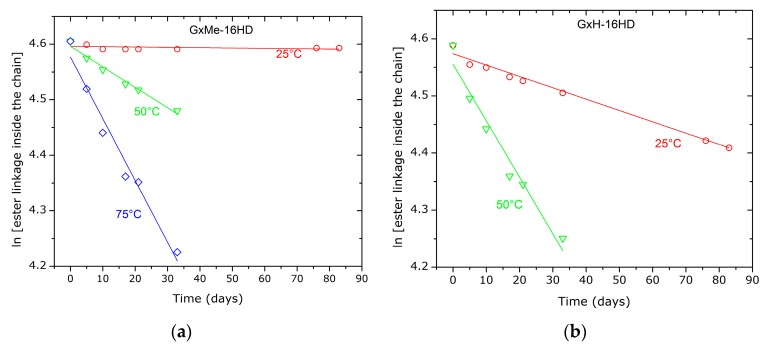
First order fit of the hydrolytic degradation GxMe-16HD and GxH-16HD samples as consequence of the treatment with citric acid 5% at 25, 50, respectively, 75 °C.

The kinetic plots of the hydrolysis show a first order fit of the reaction (Figure 11). Based on it, the calculations for the hydrolysis constant rate, at room temperature reveal that the GxH derived polyester sample despite having similar molecular weight and dispersity, are hydrolysed in acidic conditions that are approximately 30 times faster than GxMe-16HD (see Table 4). Moreover, for both types of polyester the overall hydrolysis kinetic rate shows a steep increase at temperatures well above their *T*_g_ (at 50 respectively 75 °C). 

Crystallinity and thus also the difference in the crystallisation rate can have a significant role in the hydrolytic stability and a correlation is possible i.e., between the relatively higher amount of formed crystalline regions and the better resistance to hydrolysis of the GxMe-16HD samples [39,40]. Nevertheless, it is worth mentioning that since the analysed polymer films were 0.1 mm thick, the diffusion rate through the sample is neglected. 

Accordingly, the determinant factor at structure level as also reported by Tang et al. is the presence of the pending methyl groups from the GxMe acetal, which hinders the protruding of the water molecule and thus confers a higher protection against hydrolysis; as also noted from the contact angle measurements that indicate a more hydrophobic surface of the GxMe-16HD [41].

Since the compared samples contained the same amount of catalyst, its influence was not investigated in this case. It cannot be fully excluded especially since it was reported before that for instance PET has a better thermal and hydrolytic stability when the used polycondensation catalyst was a lanthanide based one, rather than a conventional Ca acetate/Sb_2_O_3_ catalytic system [42]. 

## 4. Conclusions

The optimisation of a model transesterification reaction of two different acetalised galactarate esters, GxMe respectively GxH with an aliphatic diol was discussed. The findings reveal a difference in reactivity between the two esters. Connected to that, in the tested Sn-based catalyst concentration range, an optimum concentration of 0.3 mol % DBTO with respect to the ester was determined for the reaction with GxMe and 0.9 mol % for the one with GxH. Higher conversion to polymer was shown to happen when using a 250 mL/min N_2_ gas flow and the reaction time was decreased. 

What is generally noted is the clear influence of the type of the acetal protection group on the reactivity of the carbonyl group of the ester. When using the formaldehyde as protection group, the reaction starts slower, with a relative higher conversion at higher catalyst concentration. Thus, the acetal structure seems to determine the faster or slower activation/coordination rate of the corresponding esters with the catalyst species, and thus limiting the conversion and dispersity of the resulted polyesters.

On the structure-properties approach, the two types of acetal groups were found to induce distinct crystallisation behaviour and stability. The pending methyl groups in the GxMe acetal determine changes both in the morphology and at molecular level, and even more it contributes to a higher stability of the polyester in acidic conditions without compromising the acetal protection.

Based on these findings, a better understanding of the reactivity and properties induced by these novel sugar-based monomers was achieved. This can further lead to a better control of the synthesis of this type of polyesters and therefore of the targeted characteristics (i.e., molecular weight, crystallinity, dielectric constant, hydrolytic stability).

## Figures and Tables

**Table 1:** Overall kinetic constants determined for a second order fitting of the transesterification reactions of GxMe, respectively, GxH diester with 1,6-hexanediol and the R squared values for the linear fit.

Entry		[DBTO]					
		0.3 mol %	0.6 mol %	0.9 mol %	1 mol %	2 mol %	4 mol %
GxMe-16HD	*k_a_*′ (L/mol s) *R*^2^ *fit*	0.110 *0.996*	- -	- -	0.071 *0.974*	0.021 *0.688*	0.006 *0.344*
GxH-16HD	*k_a_*′ (L/mol s) *R*^2^ *fit*	- -	0.063 *0.938*	0.087 *0.988*	-	0.069 *0.986*	0.033 *0.910*

**Table 2:** Overview of the thermal and mechanical behaviour of the two galactarate acetalised polyesters.

Sample	*T*_g_, * °C	*T*_m_ *, °C	Melting Enthalpy ΔH *, J/g	Crystallinity **, %	Contact Angle, deg.	Elastic Modulus, GPa ***
GxMe-16HD	−3	97	19.5	44	86	0.15 ± 0.025
GxH-16HD	−8	-	-	35	75	0.1 ± 0.02

* Values determined from the second heating curve of samples obtained after synthesis and purification. ** The relative crystallinity values were calculated based on the two-phase model [33] where it is defined as $x=\frac{A_{c}}{A_{c}+K\;A_{a}}$; *A_c_* is the area of the peak corresponding to the crystalline phase, *A_a_* is the area of the peak corresponding to the amorphous phase and *K* is a constant related to different scattering factors of the two phases which (for relative measures *K* = 1). *** The values for the elastic modulus are expressed as the arithmetic mean of 7 measurements ± standard deviation.

**Table 3:** Dielectric constant of GxMe-16HD and GxH-16HD determined with an additive molar group contribution (calculation for theoretically amorphous, respectively, 50% semicrystalline samples).

Entry	Dielectric Constant	
	Amorphous	Semicrystalline
GxMe-16HD	2.74	2.67
GxH-16HD	3.24	3.15

**Table 4:** Apparent kinetic constants of the hydrolysis of GxMe-16HD respectively GxH-16HD in citric acid 5% at 25, 50 and 75 °C.

Temperature	k_hydrolysis_, 1/s	
	GxMe-16HD	GxH-16HD
25 °C	6.28 × 10^−5^	1.98 × 10^−3^
50 °C	3.69 × 10^−3^	9.9 × 10^−3^
75 °C	1.11 × 10^−2^	n.a.

## Supplementary Materials

The following are available online at http://www.mdpi.com/2073-4360/10/3/248/s1, Figure S1: 1H-NMR spectra of the GxMe-16HD and GxH-16HD polyesters (after the vacuum stage); Figure S2: DSC of the GxMe and GxH polyesters after isothermal tratment at 75 °C for 24 h; Figure S3: TGA of the GxMe and GxH esters.
