# Supplementary material for: Acetalised Galactarate Polyesters: Interplay between Chemical Structure and Polymerisation Kinetics
Source: Polymers (Basel). 2018 Feb 28;10(3):248. doi: 10.3390/polym10030248 (PMC6415047; doi:10.3390/polym10030248)
Supplement: Supplementary file 1 [file polymers-10-00248-s001.docx]

Article

Acetalised Galactarate Polyesters: Interplay between Chemical Structure and Polymerisation Kinetics

Ionela Gavrila, Patrizio Raffa and Francesco Picchioni *

Department of Chemical Engineering, ENTEG, University of Groningen, Nijenborgh 4,
9747 AG Groningen, The Netherlands; i.gavrila@rug.nl (I.G.); p.raffa@rug.nl (P.R.)

***** Correspondence: f.picchioni@rug.nl; Tel.: +31-050-363-4333

Received: 15 February 2018; Accepted: 25 February 2018; Published: 28 February 2018

**Supplementary Materials:**

**Figure S1**. 1H-NMR spectra of the GxMe-16HD and GxH-16HD polyesters (after the vacuum stage).


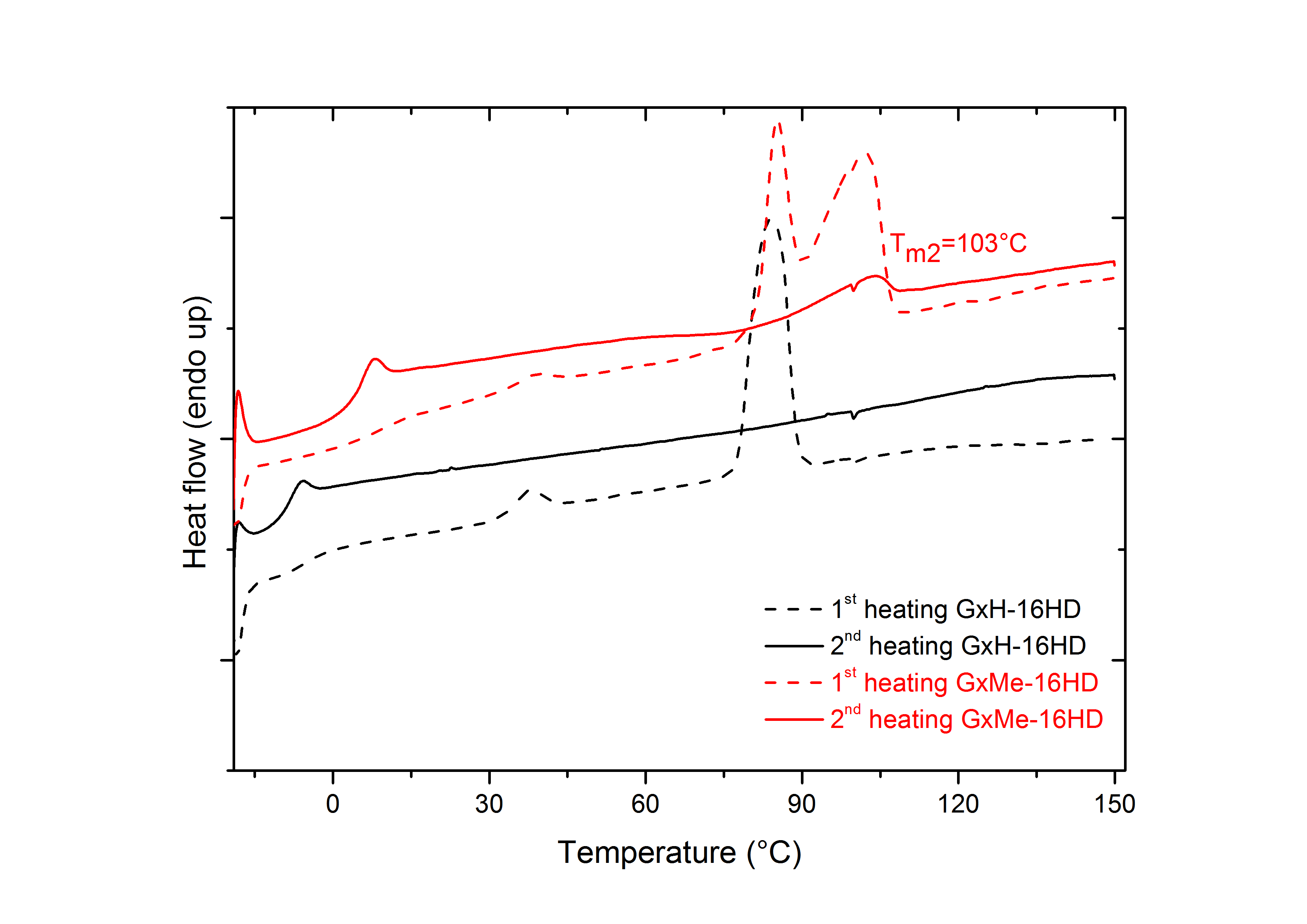


**Figure S2**. DSC of the GxMe and GxH polyesters after isothermal tratment at 75°C for 24hours.


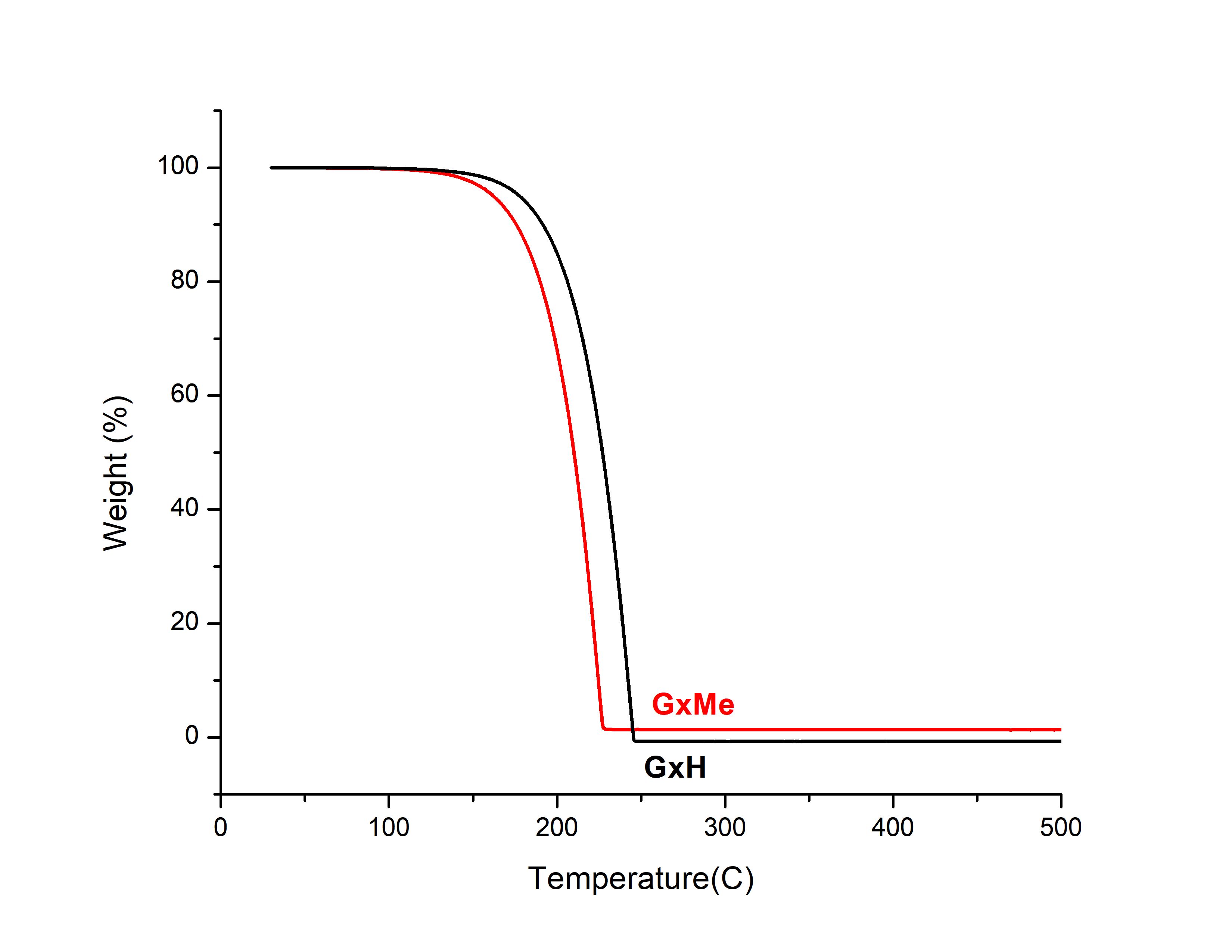


**Figure S3**. TGA of the GxMe and GxH esters.
